# Supplementary material for: Bite force data suggests relationship between acrodont tooth implantation and strong bite force
Source: PeerJ. 2020 Jun 30;8:e9468. doi: 10.7717/peerj.9468 (PMC7333653; doi:10.7717/peerj.9468)
Supplement: Supplemental Information 2 [file peerj-08-9468-s002.docx]

**Table S1.1: Reference list of peer-reviewed publications on *in vivo* voluntary bite-force performance in Lepidosauria in which species, bite force, and SVL (i.e., the metrics used in this study) are reported up. Publications that did not report the necessary metrics were not included in the dataset. Data used in this publication were collected from the following publications:**

1. Anderson, R.A., L.D. McBrayer, and A. Herrel. 2008. Bite force in vertebrates: opportunities and caveats for use of a nonpareil whole-animal performance measure. *Biological Journal of the Linnean Society* 93:709-720.
2. Baeckens, S., R. García-Roa, J. Martín, J. Ortega, K. Huyghe, and R. Van Damme. 2016. Fossorial and durophageous: implications of molluscivory for head size and bite capacity in a burrowing worm lizard. *Journal of Zoology* 301:193-205.
3. Broeckhoven, C., and P. le F.N. Mouton. 2014. Under pressure: morphological and ecological correlates of bite force in the rock-dwelling lizards *Ouroborus cataphractus* and *Karusasaurus polyzonus* (Squamata: Cordylidae). *Biological Journal of the Linnean Society* 111:823-833.
4. da Silva, J.M., A. Herrel, G.J. Measey, and K.A. Tolley. 2014. Sexual dimorphism in bite performance drives morphological variation in chameleons. *PLoS ONE* 9:e86846.
5. Edwards, J.R., and S.P. Lailvaux. 2013. Do interspecific interactions between females drive shifts in habitat use? A test using lizards *Anolis carolinensis* and *A. sagrei*. *Biological Journal of the Linnean Society* 110:843-851.
6. Edwards, S., K.A. Tolley, B. Vanhooydonck, G.J. Measey, and A. Herrel. 2013. Is dietary niche breadth linked to morphology and performance in Sandveld lizards *Nucras* (Sauria: Lacertidae). *Biological Journal of the Linnean Society* 110:674-688.
7. Fabre, A-C., D.V. Andrade, K. Huyghe, R. Cornette, and A. Herrel. 2014. Interrelationships between bones, muscles, and performance: biting in the lizard *Tupinambis merianae*. *Evolutionary Biology* 41:518-527.
8. Gowan, T.A., L.D. McBrayer, D.C. Rostal. 2010. Seasonal variation in testosterone and performance in males on a non-territorial lizard species. *Physiology & Behavior* 100:357-363.
9. Gröning, F., M.E.H. Jones, N. Curtis, A. Herrel, O. O’Higgins, S.E. Evans, and M.J. Fagan. 2013. The importance of accurate muscle modeling for biomechanical analyses: a case study with a lizard skull.  *Journal of the Royal Society Interface* 10:20130216.
10. Herrel, A., and F. De Vree. 2009. Jaw and hyolingual muscle activity patterns and bite forces in the herbivorous lizard *Uromastyx acanthinurus*. *Archives of Oral Biology* 54:772-782.
11. Herrel, A., and J.C. O’Reilly. 2005. Ontogenetic scaling of bite force in lizards and turtles. *Physiological and Biochemical Zoology* 79:31-42.
12. Herrel, A., P. Aerts, and F. De Vree. 1998. Static biting in lizards: functional morphology of the temporal ligaments. *Journal of the Zoological Society of London* 244:135-143.
13. Herrel, A., A.M. Castilla, M.K. Al-Sulaiti, and J.J. Wessels. 2013. Does large body size relax constraints on bite-force generation in lizards of the genus *Uromastyx*? *Journal of Zoology* 292:170-174.
14. Herrel, A., L. Spithoven, R. Van Damme, and F. De Vree. 1999. Sexual dimorphism of head size in *Gallotia galloti*: testing the niche divergence hypothesis by functional analyses. *Functional Ecology* 13:289-297.
15. Herrel, A., E. De Grauw, and J.A. Lemos-Espinal. 2001a. Head shape and bite performance in xenosaurid lizards. *Journal of Experimental Zoology* 290:101-107.
16. Herrel, A., R. Joachim, B. Vanhooydonck, and D.J. Irschick. 2006. Ecological consequences of ontogenetic changes in head shape and bite performance in the Jamaican lizard *Anolis lineatopus*. *Biological Journal of the Linnean Society* 89:443-454.
17. Herrel, A., K. Huyghe, B. Vanhooydonck, T. Backeljau, K. Breugelmans, I. Grbac, R. Van Damme, and D.J. Irschick. 2008. Rapid large-scale evolutionary divergence in morphology and performance associated with exploitation of a different dietary resource. *Proceedings of the National Academy of Science* 105:4792-4795.
18. Herrel, A., L.D. McBrayer, and P.M. Larson. 2007. Functional basis of sexual differences in bite force in the lizard *Anolis carolinensis*. *Biological Journal of the Linnean Society* 91:111-119.
19. Herrel, A., R. Van Damme, B. Vanhooydonck, and F. De Vree. 2001b. The implications of bite performance for diet in two species of lacertid lizards. *Canadian Journal of Zoology* 79:662-670.
20. Herrel, A., J.A. Moore, E.M. Bredeweg, and N.J. Nelson. 2010. Sexual dimorphism, body size, bite force and male mating success in tuatara. *Biological Journal of the Linnean Society* 100:287-292.
21. Husak, J.F., A.K. Lappin, S.F. Fox, and J.A. Lemos-Espinal. 2006. Bite-force performance predicts dominance in male venerable collared lizards (*Crotaphytus antiquus*). Copeia 2006:301-306.
22. Huyghe, K., A. Herrel, B. Vanhooydonck, J.J. Meyers, and D.J. Irschick. 2007a. Microhabitat use, diet, and performance data on the Hispaniolan twig anole, *Anolis sheplani*: pushing the boundaries of morphospace. *Zoology* 110:2-8.
23. Huyghe, K., B. Vanhooydonck, A. Herrel, Z. Tadić, and R. Van Damme. 2007b. Morphology, performance, behavior and ecology of three color morphs in male of the lizard *Podacris melisellensis*. *Integrative and Comparative Biology* 47:211-220.
24. Irschick, D.J., and J.J. Meyers. 2007. An analysis of the relative roles of plasticity and natural selection in the morphology and performance of a lizard (*Urosaurus ornatus*). *Oecologia* 153:489-499.
25. Jones, M.E.H., and A.K. Lappin. 2009. Bite-force performance of the last rhynchocephalian (Lepidosauria: *Sphenodon*). *Journal of the Royal Society of New Zealand* 39:71-83.
26. Lappin, A.K., P.S. Hamilton, and B.K. Sullivan. 2006. Bite-force performance and head shape in a sexually dimorphic crevice-dwelling lizard, the common chuckwalla [*Sauromalus ater* (= obesus)]. *Biological Journal of the Linnean Society* 88:215-222.
27. Lopez-Darias, M., B. Vanhooydonck, R. Cornette, and A. Herrel. 2015. Sex-specific differences in ecomorphological relationships in lizards of the genus *Gallotia*. *Functional Ecology* 29:506-514.
28. McBrayer, L.D. 2004. The relationship between skull morphology, biting performance and foraging mode in Kalahari lacertid lizards. *Zoological Journal of the Linnean Society* 140:403-416.
29. McBrayer, L.D., and R.A. Anderson. 2007. Sexual size dimorphisms and bite force in the northern alligator lizard, *Elgaria coerulea. Journal of Herpetology* 41:554-559.
30. McBrayer, L.D., and T.D. White. 2002. Bite force, behavior, and electromyography in the teiid lizard, *Tupinambis teguixin*. *Copeia* 2002:111-119.
31. McLean, C.A., and D. Stuart-Fox. 2015. Rival assessment and comparison of morphological and performance-based predictors of fighting ability in Lake Eyre dragon lizards, *Ctenophorus maculosus* 69:523-531.
32. Meyers, J.J., and D.J. Irschick. 2015. Does whole-organism performance constrain resource use? A community test with desert lizards. *Biological Journal of the Linnean Society* 115:859-868.
33. Meyers, J.J., K.C. Nishikawa, and A. Herrel. 2018. The evolution of bite force in horned lizards: the influence of dietary specialization. *Journal of Anatomy* 232:214-226.
34. Sagonas, K., P. Pafilis, P. Lymberakis, C.M. Donihue, A. Herrel, and E.D. Valakos. 2014. Insularity affects head morphology, bite force and diet in a Mediterranean lizard. *Biological Journal of the Linnean Society* 112:469-484.
35. Schaerlaeken, V., V. Holanova, R. Boistel, P. Aerts, P. Velensky, I. Rehak, D.V. Andrade, and A. Herrel. 2012. Built to bite: feeding kinematics, bite forces, and head shape of a specialized durophagous lizard, *Dracaena guianensis* (Teiidae). *Journal of Integrative Biology* 317A:371-381.
36. Segall, M., K.A. Tolley, B. Vanhooydonck, G.J. Measey, and A. Herrel. 2013. Impact of temperature on performance in two species of South African dwarf chameleon, *Bradypodoin pumilum* and *B. occidentale*. *The Journal of Experimental Biology* 216:3828-3836.
37. Simon, M.N., R. Brandt, T. Kohlsdorf, and S.J. Arnold. 2019. Bite performance surfaces of three ecologically divergent Iguanidae lizards: relationships with lower jaw bones. *Biological Journal of the Linnean Society* 127:810-825.
38. Vanhooydonck, B., F.B. Cruz, C.S. Abdala, D.L.M. Azócar, M.F. Bonino, and A. Herrel. 2010. Sex-specific evolution of bite performance in *Liolaemus* lizards (Iguania: Liolaemidae): the battle of the sexes. *Biological Journal of the Linnean Society* 101:461-475.
39. Verwaijen, D., R. Van Damme, and A. Herrel. 2002. Relationships between head size, bite force, prey handling efficiency and diet in two sympatric lacertid lizards. *Functional Ecology* 16:842-850.

**Table S1.2: Reference list of peer-reviewed publications on bite-force performance in Lepidosauria, and includes publications that record *in vivo* and/or modeled bite force measurements. The following papers did not report raw bite force data metrics included in our analyses, but are included in the histogram of families of lepidosaurs who have had their bite forces measured. All references in Table S1 were are also included in the histogram.**

1. Baeckens, S., T. Driessens, K. Huyghe, and B. Vanhooydonck, and R. Van Damme. 2018. Intraspecific variation in the formation content of an ornament: why relative dewlap size signals bite force in some, but not all island populations of *Anolis sagrei*. *Integrative and Comparative Biology* 58:25-37.
2. Baeckens, S., D. Llusia, R. García-Roa, and J. Martín. 2019. Lizard calls convey honest information on body size and bite performance: a role in predator deterrence? *Behavioral Ecology and Sociobiology* 73:87
3. Baxter-Gilbert, J.H., and M.J. Whiting. 2019. Street fighters: bite force, injury rates, and density of urban Australian water dragons (*Intellagama lesueurii*). *Austral Ecology* 44:255-264.
4. Cameron, S.F., M.L. Wynn, and R.S. Wilson. 2013. Sex-specific trade-offs and compensatory mechanisms: bite force and sprint speed pose conflicting demands on the design of geckos (*Hemidactylus frenatus*). *The Journal of Experimental Biology* 216:3781-3789.
5. Chen, J., Y. Qi, Y. Wu, and Y. Tang. 2019. Covariations between personality behaviors and metabolic/performance traits in an Asian agamid lizard (*Phyrnocephalus vlangalii*). *PeerJ* 7:e7205
6. D’Amore, D.C., K. Moreno, C.R. McHenry, and S. Wroe. 2011. The effects of biting and pulling on the forces generated during feeding in the Komodo Dragon (*Varanus komodoensis*). PLoS ONE 6:e26226.
7. D’Amore, D.C., D. Meadows, S. Clulow, J.S. Doody, D. Rhind, and C.R. McHenry. 2018. Increasing dietary breadth through allometry: bite forces in sympatric Australian skinks. *Herpetology Notes* 11:179-187.
8. de Meyer, J., D.J. Irschick, B. Vanhooydonck, J.B. Losos, D. Adriaens, and A. Herrel. 2019. The role of bite force in the evolution of head shape and head shape dimorphism in *Anolis* lizards. *Functional Ecology* 33:2191-2202.
9. Des Roches, S., L.J. Harmon, and E.B. Rosenblum. 2015. Colonization of a novel depauperate habitat leads to trophic niche shifts in three desert lizard species. *Oikos* 125: 343-353.
10. Donihue, C.M. 2016. Aegean wall lizards switch foraging modes, diet, and morphology in a human-built environment. *Ecology and Evolution* 6:7433-7442.
11. Donihue, C.M., K.M. Brock, J. Foufopoulos, and A. Herrel. 2016. Feed or fight: testing the impact of food availability and intraspecific aggression on the functional ecology of an island lizard. *Functional Ecology* 30:566-575.
12. Dufour, C.M.S., J.B. Losos, and A. Herrel. 2018. Do differences in bite force and head morphology between a native and an introduced species of anole influence the outcome of species interactions? *Biological Journal of the Linnean Society* 125:576-585.
13. Edwards, S., A. Herrel, B. Vanhooydonck, and G.J. Measey, and K.A. Tolley. 2016. Diving in head first: trade-offs between phenotypic traits and sand-diving predator escape strategy in *Meroles* desert lizards
14. Henningsen, J.P., and D.J. Irschick. 2012. An experimental test of the effect of signal size and performance capacity on dominance in the green anole lizard. *Functional Ecology* 26:3-10.
15. Herrel, A., and V. Holanova. 2008. Cranial morphology and bite force in *Chamaeleolis* lizards – adaptations to mollscivory. *Zoology* 11:467-475.
16. Herrel, A., D.V. Andrade, J.E. de Carvalho, A. Brito, A. Abe, and C. Navas. 2009. Aggressive behavior and performance in the tegu lizard *Tupinambis merianae*. *Physiological and Biochemical Zoology* 82:680-685.
17. Herrel, A., M. Lopez-Darias, B. Vanhooydonck, R. Cornette, T. Kohlsorf, and R. Brandt. 2016. Do adult phenotypes reflect selection on juvenile performance? A comparative study on performance and morphology in lizards. *Integrative and Comparative Biology* 56:469-478.
18. Huyghe, K., A. Herrel, D. Adriaens, Z. Tadić, and R. Van Damme. 2009. It is all in the head: morphological basis for differences in bite force among colour morphs of the Dalmatian wall lizard. *Biological Journal of the Linnean Society* 96:13-22.
19. Huyghe, K., L.M. San-Jose, M.P. Alcázar, and P.S. Fitze. 2013. An ecomorphological analysis of the determinants of mating success. *Biological Journal of the Linnean Society* 110:658-664.
20. Huyghe, K., J.F. Husak, I.T. Moore, B. Vanhooydonck, R. Van Damme, M. Molina-Borja, and A. Herrel. 2010. Effects of testosterone on morphology, performance and muscle mass in a lizard. *Journal of Experimental Zoology* 313A:9-16.
21. Huyghe, K., R. Van Damme, K. Breugelmans, A. Herrel, B. Vanhooydonck, Z. Tadič, and T. Backeljau. *Behavioral Ecology and Sociobiology* 68:1357-1366.
22. Husak, J.F., A.K. Lappin, and R.A. Van den Bussche. 2009. The fitness advantage of a high-performance weapon. *Biological Journal of the Linnean Society* 96:840-845.
23. Irschick, D.J., M. Ramos, C. Buckley, J. Elstrott, E. Carlisle, S.P. Lailvaux, N. Bloch, A. Herrel, and B. Vanhooydonck. 2006. Are morphology-performance relationships invariant across different seasons? A test with the green anole lizard (*Anolis carolinensis*). *Oikos* 114:49-59.
24. Kaliontozopoulou, A., D.C. Adams, A. van der Meijden, A. Perera, and M.A. Carretero. 2012. Relationships between head morphology, bite performance and ecology in two species of *Podarcis* wall lizards. *Evolutionary Ecology* 26:825-845.
25. Lailvaux, S.P., and D.J. Irschick. 2006. No evidence for female association with high-performance males in the green anole lizard, *Anolis carolinensis*. *Ethology* 112:707-715.
26. Lailvaux, S.P., and D.J. Irschick. 2007. The evolution of performance-based male fighting ability in Caribbean *Anolis* lizards. *The American Naturalist* 170:573-586.
27. Lailvaux, S.P., A. Herrel, B. VanHooydonck, J.J. Meyers, and D.J. Irschick. 2005. Performance capacity, fighting tactics and the evolution of life-stage male morphs in the green anole lizard (*Anolis carolinensis*). *Proceedings of the Royal Society of London B* 291:2501-2508.
28. Lailvaux, S.P., A.M. Cespedes, W.D. Weber, and J.F. Husak. 2019. Sprint speed is unaffected by dietary manipulation in trained male *Anolis carolinensis* lizards. *Journal of Experimental Zoology* 2019:jez2338.
29. Lappin, A.K., and J.F. Husak. 2005. Weapon performance, not size, determines mating success and potential reproductive output in the collared lizard (*Crotaphytus collaris*). 166:426-436.
30. Lappin, A.K., and M.E.H. Jones. 2014. Reliable quantification of bite-force performance requires use of appropriate biting subtrate and standardization of bite out-lever. *The Journal of Experimental Biology* 217:4303-4312.
31. Lappin, A.K., Y. Brandt, J.F. Husak, J.M. Macedonia, and D.J. Kemp. 2006. Gaping displays reveal and amplify a mechanically based index of weapon performance. *The American Naturalist* 168:100-113.
32. MacGregor, H.E.A., G.M. White, and T. Uller. 2017. Comparison of reproductive investment in native and non-native populations of common wall lizards reveals sex differences in adaptive potential. *Oikos* 126:1564-1574.
33. Measey, G.J., K. Hopkins, and K.A. Tolley. 2009. Morphology, ornaments and performance in two chameleon ecomorphs: is the casque bigger than the bite. *Zoology* 112:217-226.
34. Measey, G.J., A.D. Rebelo, A. Herrel, B. Vanhooydonck, and K.A. Tolley. 2011. Diet, morphology and performance in two chameleon morphs: do harder bites equate with harder prey. *Journal of Zoology* 285:247-255.
35. Meyers, J.J., A. Herrel, and J. Birch. 2002. Scaling of morphology, bite force and feeding kinematics in an iguanian and scleroglossan lizard. In: Aerts, P., K. D’Août, A. Herrel, and R. Van Damme (eds.) *Topics in Functional and Ecological Vertebrate Morphology*. Shaker Publishing, Maastricht, Germany. Pp. 47-62.
36. Nobel, D.W.A., K.V. Fanson, and M.J. Whiting. 2014. Sex, androgens, and whole-organism performance in an Australian lizard. *Biological Journal of the Linnean Society* 111:834-849.
37. Porro, L.B., C.F. Ross, J. Iriarte-Diaz, J.C. O’Reilly, S.E. Evans, and M.J. Fagan. 2014. *In vivo* cranial bone strain and bite force in the agamid lizard *Uromastyx geyri*. *The Journal of Experimental Biology* 217:1983-1992.
38. Ross, C.F., L.B. Porro, A. Herrel, S.E. Evans, and M.J. Fagan. 2018. Bite force and cranial bone strain in four species of lizards. *Journal of Experimental Biology* 221:jeb180240
39. Schaerlaeken, V., A. Herrel, P. Aerts, and C.F. Ross. 2008. The functional significance of the lower temporal bar in *Sphenodon punctatus*. *The Journal of Experimental Biology* 211:3908-3914.
40. Tseng, H.-Y., C.-P. Liao, J.-Y. Wang, and W.-S. Huang. 2018. Parental behavior drives large bite force in an insular skink population. *Journal of Zoology* 307:223-231.
41. Vanhooydonck, B., R. Boistel, V. Fernandez, and A. Herrel. 2011. Push and bite: trade-offs between burrowing and biting in a burrowing skink (*Acontias percivali*). *Biological Journal of the Linnean Society* 102:91-99.
42. Vanhooydonck, B., A.Y. Herrel, R. Van Damme, and D.J. Irschick. 2005a. Does dewlap size predict male bite performance in Jamaican *Anolis* lizards? *Functional Ecology* 19:38-42.
43. Vanhooydonck, B., A. Herrel, R. Van Damme, J.J. Meyers, and D.J. Irschick. 2005b. The relationship between dewlap size and performance changes with age and sex in a green anole *(Anolis carolinensis*) lizard population. *Behavioral Ecology and Sociobiology* 59:157-165.
44. Wittorski, A., J.B. Losos, and A. Herrel. 2016. Proximate determinants of bite force in *Anolis* lizards. *Journal of Anatomy* 228:85-95.
45. Yewers, M.S.C., T.S. Jessop, and D. Stuart-Fox. 2017. Endocrine differences among colour morphs in a lizard with alternative behavioural strategies. *Hormones and Behavior* 93:118-127.
46. Žagar, A., M.A. Cerretero, A. Vrezec, K. Drašler, and A. Kaliontzopoulou. 2017. Towards a functional understanding of species coexistence: ecomorphological variation in relation to whole-organism performance in two sympatric lizards. *Functional Ecology* 31:1780-1791.
